# Risikokommunikation von Behörden – Herausforderungen und Perspektiven

**DOI:** 10.1007/s00103-022-03531-0

**Published:** 2022-05-03

**Authors:** Suzan Fiack, Joseph Kuhn, Wolfgang Straff

**Affiliations:** 1 Bundesinstitut für Risikobewertung, Max-Dohrn-Str. 8–10, 10589 Berlin, Deutschland; 2 Bayerisches Landesamt für Gesundheit und Lebensmittelsicherheit, Veterinärstr. 2, 85764 Oberschleißheim, Deutschland; 3 Umweltbundesamt, Corrensplatz 1, 14195 Berlin, Deutschland

Risikogruppen, Risikofaktoren, Risikogebiete – in der Coronapandemie ist der Begriff „Risiko“ mit Wucht in den Alltag der Bevölkerung eingedrungen und hat das Bewusstsein von Millionen Menschen bestimmt. Zugleich hat die Pandemie eindringlich gezeigt, dass wir uns als Individuen und als Gesellschaft immer wieder mit neuen Herausforderungen auseinandersetzen und sie bewältigen müssen. Von entscheidender Bedeutung ist dabei, wie Risiken wahrgenommen und wie sie kommuniziert werden, seitens der Bürgerinnen und Bürger ebenso wie seitens der Politik und der Behörden.

Gefahren sind im Leben allgegenwärtig, ja unvermeidlich. Zukunft birgt stets beides, Chancen wie Risiken. Dabei ist es für moderne Gesellschaften kennzeichnend, dass viele ihrer Risiken „hausgemacht“ sind, ein Produkt unserer Zivilisation. Darauf zielte der Begriff der *Risikogesellschaft*, wie der einprägsame Titel des 1986 erschienenen Buchs von Ulrich Beck lautete. Der Klimawandel und die Coronapandemie machen deutlich, dass Technik und Natur auf vielfältige Weise miteinander verwoben sind. Auch kriegerische Auseinandersetzungen beinhalten Risiken, auf die Behörden und andere Institutionen reagieren müssen. Ganz abgesehen davon, dass auch in modernen Gesellschaften die natürlichen Risiken immer noch höchst präsent sind. Erdbeben, Tsunamis oder Vulkanausbrüche gab es, gibt es und wird es immer geben. Eine wachsende Weltbevölkerung wird auch sie zu meistern haben.

Es hängt sehr vom spezifischen Risiko ab, wie es wahrgenommen und bewältigt wird – und wie darüber informiert und kommuniziert wird. Die Forschung dazu hat wichtige Erkenntnisse geliefert. So werden schlagartig auftretende Ereignisse wie ein Chemieunfall oder eine Reaktorhavarie intensiver wahrgenommen als schleichende Entwicklungen. Wir gehen gedanklich mit Risiken, die wir glauben kontrollieren zu können, wie dem Unfallrisiko beim Autofahren, anders um als mit solchen, denen wir uns hilflos ausgeliefert fühlen, wie der Belastung unserer Lebensmittel mit unerwünschten Stoffen. Und wir bewerten unsichtbare Gefahren wie Viren, Feinstaub oder Strahlen anders als sichtbare. Dementsprechend unterschiedlich wird über solche Risiken in der Gesellschaft kommuniziert – mal distanziert und unterkühlt, mal aufgeregt und empört.

Dazu kommt, dass in manchen traditionellen Medien, vor allem aber in den sozialen Medien die Tendenz spürbar ist, möglichst knappe Aussagen über ein gesundheitliches Problem oder Risiko zu treffen. Durch solche verkürzten Darstellungen, Twitter-Botschaften oder Schlagzeilen für das Handyformat kann es dann sowohl bei den „Informationssendern“ als auch bei den „Empfängern“ zu Missverständnissen rund um Risiken und Gefährdungen kommen. Zusätzlich kann die Tendenz, nach Bestätigung für die eigenen Ansichten und Vorlieben zu suchen, zusammen mit Besorgnis oder Ängsten bei vielen Nutzerinnen und Nutzern sozialer Medien zu einer unzureichenden Aufklärung über die tatsächlichen Risiken führen. Hinzu kommt auch das Phänomen der „Filterblasen“, welche durch spezifische Algorithmen des sozialen Mediums bestimmte Inhalte hervorheben. In der Coronapandemie wurde das besonders deutlich. Fehlinformationen, Desinformation und Verschwörungserzählungen haben zeitweise das Vertrauen in die Infektionsschutzmaßnahmen bedroht. Die Risikokommunikation seitens Medien, Politik und Behörden stand vor der Herausforderung, sowohl Orientierung zu geben als auch wissenschaftliche Unsicherheiten zu thematisieren. Solange wissenschaftliche Evidenz schwach ist, ist eine konsistente, klare und Orientierung gebende Kommunikation schwierig. Gleichwohl wird genau das erwartet, zumal Krisen stets auch die Politik und die Behörden unter Druck setzen.

Nachsteuerungen oder Änderungen der gesundheitlichen Empfehlungen oder der behördlichen Vorgaben werden von manchen Menschen nicht als erforderliche Anpassung an die wissenschaftliche Evidenzlage, sondern mitunter als Zumutung oder als „politische Maßnahme“ empfunden. Die immer wieder, z. B. auch in der WHO-Leitlinie „Communicating risk in public health emergencies“ [1], geäußerte Empfehlung, wissenschaftliche Unsicherheit offen und verständlich zu kommunizieren, ist vor diesem Hintergrund eine besondere Herausforderung.

Wie also kann die Kommunikation gesundheitlicher Risiken gelingen, insbesondere was die Risikokommunikation von Behörden angeht? Wie kann sie die Risikoeinschätzung der Menschen unterstützen? Welche Rolle spielt die Risikowahrnehmung? Erhöht die Kommunikation wissenschaftlicher Unsicherheit das Vertrauen oder untergräbt sie es? Welche Rolle spielen neue Formate und Trends wie die sozialen Medien? Welche Formate sind für welches Ziel angemessen?

Im Januar 2022 hat der Corona-ExpertInnenrat der Bundesregierung seine Empfehlungen „Zur Notwendigkeit evidenzbasierter Risiko- und Gesundheitskommunikation“ [2] veröffentlicht. Er resümiert: „Die Corona-Pandemie ist nur eine von mehreren kollektiven und globalen Gesundheitskrisen, auf die die Gesellschaft reagieren muss. Deshalb bedarf es der Einrichtung einer nachhaltigen Infrastruktur, um die Bevölkerung evidenzbasiert, schnell und effektiv zu informieren und in ihrer Risiko- und Handlungskompetenz zu unterstützen.“ Möglicherweise können dabei neben der Risiko- und Kommunikationsforschung auch die Erfahrungen von Behörden mit unterschiedlichen Kommunikationskonzepten weiterführen.

Anlass genug also, das Thema Risiko- und Krisenkommunikation wieder einmal auf die Agenda des Bundesgesundheitsblatts zu setzen, nachdem es dazu bereits wiederholt Beiträge gab, z. B. in den Heften 1 und 3/2021. Die vorliegende Ausgabe versammelt dazu Bestandsaufnahmen und aktuelle Forschungsergebnisse.

Wir wünschen eine gewinnbringende Lektüre.
